# CD44/CD24 Expression in recurrent gastric cancer: a retrospective analysis

**DOI:** 10.1186/1471-230X-12-95

**Published:** 2012-07-28

**Authors:** Ching-Shya Yong, Chih-Ming Ou Yang, Yenn-Hwei Chou, Chao-Sheng Liao, Chung-Wei Lee, Chin-Cheng Lee

**Affiliations:** 1 Department of Surgery, Shin Kong Wu Ho-Su Memorial Hospital, No.95, Wen Chang Road, Shih Lin District, Taipei City, 11120, Taiwan; 2 Department of Biological Engineering, Massachusetts Institute of Technology, Massachusetts, USA; 3 Department of Pathology, Shin Kong Wu Ho-Su Memorial Hospital, No.95, Wen Chang Road, Shih Lin District, Taipei City, 11120, Taiwan

**Keywords:** CD44, CD24, Prognosis, Recurrent gastric cancer

## Abstract

**Background:**

To correlate CD44/CD24 expression with gastric cancer recurrence and prognosis. Gastric cancer is the second leading cause of cancer mortality due to the high recurrence rate, of which the molecular signature has not yet been identified.

**Methods:**

We retrospectively reviewed the hospital records of patients with gastric cancer. Among 500 patients receiving curative resection, 95 patients had recurrence. Twenty patients from the recurrence group (95 patients) and 20 patients from the non-recurrence group (405 patients) were randomly selected and identified as “study” and “control” groups, respectively. We reviewed patients’ histological study of CD44/CD24 expression by performing immunohistochemistry and recurrence rate.

**Results:**

Study group had higher TNM stage (III-IV) than control group (80% vs. 25%, *P* = 0.001). Proportion of lymph node metastasis was significantly higher in study group than that in control group (90% vs. 45%, *P* = 0.002), and proportion of patients with 5 or more metastatic lymph nodes was also significantly higher in study group than in control group (45% vs. 15%, *P* = 0.007). Univariate analysis revealed no difference in risk of gastric cancer recurrence between CD44+ and CD44- patients (OR = 1.00, 95% CI: 0.29-3.45, *P* =1.000). CD24+ patients showed no greater significance of gastric cancer recurrence than CD24- patients (OR = 1.86, 95% CI: 0.52-6.61, *P* = 0.339). After adjusting for other risk factors, the association of CD44 expression (aOR = 0.66, 95% CI: 0.10-4.26, *P* = 0.658), CD24 expression (aOR = 0.09, 95% CI: 0.01-1.35, *P* = 0.081) or combined (CD44/CD24) with gastric cancer recurrence were not significant.

**Conclusion:**

Neither individual expression of CD24 or CD44, nor combined expression of CD44/CD24 was associated with recurrence of gastric carcinoma.

## Background

Gastric cancer (GC) is the fifth leading cause of cancer death in Taiwan, although its incidence and mortality rate have been declining in the past five decades. Globally, GC is fourth most common among all types of cancer diagnoses, and is the second leading cause of cancer mortality [1], despite improvements in surgical techniques and development of new chemotherapeutic regimens. Annual deaths have reached 700,000 worldwide and 42% are reported in China alone [1]. Even after curative resection, 40% of patients with advanced gastric cancer die of recurrence [2]. The prognosis for patients after curative surgery remains poor due to the high recurrence rate. The overall 5-year survival rate for patients who undergo curative surgical resection for gastric carcinoma ranges from 47% to 60.4%, and the recurrence rate ranges from 15.4% to 37% [3].

Genetic susceptibility variants and molecular alterations related to environmental and lifestyle factors are known to contribute to development of GC, but even though many studies have investigated molecular markers for the disease, the true mechanisms of GC carcinogenesis remain obscure [3]. Recurrence mechanisms also lack definitive explanation [4]. Even though we know gastric carcinoma is prone to recur despite curative resection, no molecular biomarker is currently available to predict gastric carcinoma recurrence after resection. While clinical predictive factors, such as tumor staging, can predict recurrence of advanced gastric cancer and are well recognized as essential predictors of prognosis, is there any molecular-based biomarker that can serve as a useful predictor for recurrence of advanced gastric cancer after curative resection (R0 resection)?

Both CD44 and CD24 are known to contribute to cellular signaling and cell adhesion, and their role in cancer recurrence has been investigated. In a review of existing literature on the role of CD44/CD24 in recurrent human cancer, investigators showed positive associations between CD44+/CD24- and prognosis, especially in breast cancer; and the CD44+/CD24- phenotype of breast cancer cells was also associated with invasive properties. [5] CD44 and CD24 have been shown to regulate invasion and metastasis of breast cancer cells either positively or negatively. Tumorigenic breast cancer cells that express high levels of CD44 and low or undetectable levels of CD24 (CD44+/CD24^-/low^) may be resistant to chemotherapy and therefore responsible for cancer relapse [6]. CD44 was also highly expressed in gastric adenocarcinoma and its expression correlated with poor prognosis in patients with the intestinal type of gastric adenocarcinoma [7]. Based on the implications of these previous studies, we hypothesized that CD44+/CD24- expression might be correlated with gastric cancer recurrence.

To our knowledge, current studies report no valid adhesion molecule to predict disease recurrence after patients undergo curative resection for gastric carcinoma. Because the significance of CD44+/CD24- is probably not unique to breast cancer, and CD44 has been highly expressed in gastric cancer, we might speculate that similar or other molecules may also regulate the process of recurrence for gastric cancer. Therefore, we decided to investigate the expression of adhesion molecule CD44/CD24 in recurrent gastric cancer and its possible predictive relevance in future clinical practice. The purpose of this study was to evaluate the correlation of CD44/CD24 expression with recurrent gastric cancer and to determine its prognostic significance.

## Methods

### Patient selection

The protocol for this study was reviewed and approved by the internal review board of Shin Kong Memorial Hospital. Data were obtained from a retrospectively maintained database consisting of patients evaluated for gastric cancer from 1993 to 2007. Tumors were staged according to the criteria of the American Joint Commission for Cancer (AJCC 6^th^ edition) for gastric cancer and were classified histologically according to the WHO criteria; Lauren’s classification was also applied (intestinal type GC corresponds to well- or moderately differentiated tumors; diffuse type corresponds with poorly differentiated tumors). A retrospective review of patients’ medical records was also completed and data were collected for age, sex, and final pathologic diagnosis.

During the period from January 1993 to December 2007, 500 patients received curative resection for gastric cancer at the Department of Surgery of Shin-Kong Wu Ho-Su Memorial Hospital. Among these, 95 patients (19%) developed a recurrence during long-term follow-up.

Forty patients with gastric cancer were randomly selected as subjects in our study. We retrospectively analyzed CD44/CD24 expression in patients’ post-operative pathologic specimens. The patients were divided into two groups; 20 patients with recurrent gastric cancer were defined as the study group and the other 20 patients without recurrent gastric cancer were categorized as the control group. Among patients in the non-recurrence control group, the minimum disease-free survival was 4 years (2002–2006) and the maximum disease-free survival was 18 years (1994–2012). A total of 14 patients from the 20 non-recurrence group are still living today. All included patients provided signed informed consent to participate in the study.

### Tissue preparation

**Figure 1:**
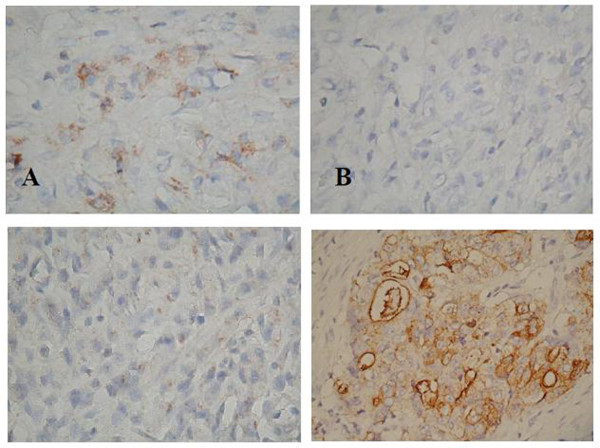
**Immunohistochemical staining of C44/CD24 protein expression in recurrent gastric cancer tissue (400x).A**: CD44 2+, **B**: CD24 -, **C**: CD44 -, **D**: CD24 3+.

Immunohistochemistry stains for CD44 and CD24 were performed for all specimens. The cancer tissues were fixed with formalin and embedded in paraffin. After paraffin removal and rehydration, antigen retrieval was performed by placing sections into a beaker containing adequate amounts of citrate buffer (pH 6.0), then heating in pressure cooker for 10 min and cooling to room temperature. After 10 min, 3% H2O2 was added. Primary antibody and secondary antibody (Envision Detection Kit, Wonderful Life Science Co. Ltd, Taiwan) were added after 60 minues and 90 minutes, respectively, coupled with PBS washing in between the addition of antibodies. DAB substrate solution was subsequently added and washed with running water before counterstaining with hematoxylin. Finally, tissues were washed with distilled water, dehydrated and mounted for microscopic evaluation. The intensity (0, 1+, 2+, 3+) of tumor cell staining was independently evaluated by two pathologists, and discrepant results were resolved by reviewing the cases together and agreeing on the scores. A complete negative staining was scored as negative (0). A weak staining (1+) was defined as minimal, but unequivocal staining in less than 10% of tumor cell. Stronger or more extensive staining was scored as moderately/strongly positive (2+/3+) (Figure 1). Tumors with weak and moderate/strong staining were defined as having positive expression, while those with negative staining only were defined as negative expression.

### Statistical analysis

Data were presented as mean ± SD for continuous variables, and frequencies with percentages for categorical variables. Differences between the study group (recurrence) and the control group (non-recurrence) were analyzed using independent *t*-test test for continuous variables, and Chi-square test or Fisher’s exact test for categorical variables as appropriate. To investigate the association of CD44 and CD24 expression as well as other risk factors with gastric cancer recurrence, the point estimates and 95% confidence intervals (CIs) of odds ratios (ORs) were calculated by univariate and multivariate logistic regression models. Multivariate logistic regression with backward selection was applied, wherein variables that did not improve the model fit at *P*<0.05 were discarded; however, CD44 and CD24 expression as well as age were always forced into the model. Two multivariate models were applied to evaluate the association of CD44 and CD24 expression with gastric cancer recurrence: Model 1 considered CD44 and CD 24 as two variables, while Model 2 considered the combined CD44/CD24 expression as one variable. All statistical analyses were performed with SAS software version 9.2 (SAS Institute Inc., Cary, NC, USA). A two-tailed P<0.05 indicated statistical significance.

## Results

A total of 40 patients were enrolled in this study, including 19 males and 21 females with mean age of 68.4 ± 11.4 years old (ranging from 44 to 88 years old). Comparison of demographic and clinical characteristics between the study group and the control group are shown in Table 1. Study group subjects were more likely to be classified as higher TNM stage (III-IV) than control group subjects (80% vs. 25%, *P* = 0.001). In addition, the proportion of lymph node metastasis was significantly higher in the study group than that in the control group (90% vs. 45%, *P* = 0.002). Moreover, the proportion of patients with five or more metastatic lymph nodes was also significantly higher in the study group than in the control group (45% vs. 15%, *P* = 0.007). No significant differences were found in other characteristics between the study group and the control group. (Table 1).

**Table 1:** Comparison of demographic and clinical characteristics between patients with / without gastric cancer recurrence

**Characteristics**	**Study Group**	**Control Group**	** *P* -value**
	**(n = 20)**	**(n = 20)**	
	**Recurrence**	**Non-recurrence**	
Gender, n(%)			
Male	12 (60.0)	7 (35.0)	0.113†
Female	8 (40.0)	13 (65.0)	
Age (years)			
mean ± SD	71.2 ± 10.6	65.7 ± 11.7	0.128‡
Age group, n(%)			
≤65	6 (30.0)	10 (50.0)	0.197†
>65	14 (70.0)	10 (50.0)	
Histology, n(%)			
Moderately differentiated (intestinal type)	9 (45.0)	6 (30.0)	0.327†
Poorly differentiated (diffuse type)	11 (55.0)	14 (70.0)	
Tumor location, n(%)			
Upper third of stomach	5 (25.0)	10 (50.0)	0.191¶
Middle third of stomach	1 (5.0)	0 (0.0)	
Lower third of stomach	14 (70.0)	10 (50.0)	
TNM Stage, n(%)			
I-II^a^	4 (20.0)	15 (75.0)	0.001*†
III-IV	16 (80.0)	5 (25.0)	
Lymph node metastasis, n(%)			
No	2 (10.0)	11 (55.0)	0.002*†
Yes	18 (90.0)	9 (45.0)	
Number of lymph node metastasis, n(%)			
0	2 (10.0)	11 (55.0)	0.007*†
1-4 s	9 (45.0)	6 (30.0)	
≥5	9 (45.0)	3 (15.0)	
CD44 expression, n(%)			
CD44-	10 (50.0)	10 (50.0)	1.000†
CD44+	10 (50.0)	10 (50.0)	
CD24 expression, n(%)			
CD24-	10 (50.0)	13 (65.0)	0.337†
CD24+	10 (50.0)	7 (35.0)	
CD44 / CD24 expression, n(%)			
CD44-/CD24-	5 (25.0)	8 (40.0)	0.622¶
CD44-/CD24+	5 (25.0)	2 (10.0)	
CD44+/CD24-	5 (25.0)	5 (25.0)	
CD44+/CD24+	5 (25.0)	5 (25.0)	

^a^ Two control patients with TNM Stage 0 were grouped into Stage I-II.

*P<0.05.

† Chi-square test; ‡ independent *t*-test; ¶Fisher’s exact test.

### Univariate and multivariate regression analysis

The univariate and multivariate associations of CD44 and CD24 expression, as well as other risk factors with gastric cancer recurrence, are shown in Table 2. In univariate analysis, patients with CD44+ showed no difference in risk of gastric cancer recurrence compared to those who were CD44- (OR = 1.00, 95% CI: 0.29-3.45, *P* = 1.000). Compared to patients who were CD24-, those with CD24+ had higher likelihood to have gastric cancer recurrence but without significance (OR = 1.86, 95% CI: 0.52-6.61, *P* = 0.339). Compared to patients with combined expression of CD44-/CD24-, the unadjusted ORs for CD44-/CD24+, CD44+/CD24-, and CD44+/CD24+ were 4.00 (95% CI: 0.55-29.10), 1.60 (95% CI: 0.30-8.49), and 1.60 (95% CI: 0.30-8.49), respectively. No significance was shown in the crude association of CD44/CD24 expression with gastric cancer recurrence. Also, higher TNM stage (III-IV), lymph node metastasis and higher numbers of metastasis lymph nodes, were all significantly associated with gastric cancer recurrence.

**Table 2:** Univariate and multivariate associations of CD44/CD24 expression and other risk factors with gastric cancer recurrence

**Characteristics**	**Recurrence rate**	**Univariate**	** *P* -value**	**Model 1†**	** *P* -value**	**Model 2‡**	** *P* -value**
		**OR (95%CI)**		**aOR (95%CI)**		**aOR (95%CI)**	
CD44 expression							
CD44-	50.0% (10/20)	1.00 (reference)	--	1.00 (reference)	--		
CD44+	50.0% (10/20)	1.00 (0.29-3.45)	1.000	0.66 (0.10-4.26)	0.658		
CD24 expression							
CD24-	43.5% (10/23)	1.00 (reference)	--	1.00 (reference)	--		
CD24+	58.8% (10/17)	1.86 (0.52-6.61)	0.339	0.09 (0.01-1.35)	0.081		
CD44 / CD24 expression							
CD44-/CD24-	38.5% (5/13)	1.00 (reference)	--			1.00 (reference)	--
CD44-/CD24+	71.4% (5/7)	4.00 (0.55-29.10)	0.171			0.60 (0.03-10.67)	0.731
CD44+/CD24-	50.0% (5/10)	1.60 (0.30-8.49)	0.581			1.61 (0.15-17.36)	0.695
CD44+/CD24+	50.0% (5/10)	1.60 (0.30-8.49)	0.581			0.06 (0.002-2.31)	0.131
Gender							
Male	63.2% (12/19)	2.79 (0.77-10.04)	0.117	39.64 (1.85-848.44)	0.019*	21.59 (1.24-377.26)	0.035*
Female	38.1% (8/21)	1.00 (reference)	--	1.00 (reference)	--	1.00 (reference)	--
Age (years)							
≤65	37.5% (6/16)	1.00 (reference)	--	1.00 (reference)	--	1.00 (reference)	--
>65	58.3% (14/24)	2.33 (0.64-8.54)	0.201	7.77 (0.89-67.99)	0.064	4.07 (0.49-34.16)	0.196
Histology							
Moderately differentiated (intestinal type)	60.0% (9/15)	1.91 (0.52-7.01)	0.330				
Poorly differentiated (diffuse type)	44.0% (11/25)	1.00 (reference)	--				
Tumor location							
Upper third + Middle third	37.5% (6/16)	1.00 (reference)	--				
Lower third	58.3% (14/24)	2.33 (0.64-8.54)	0.201				
TNM Stage							
I-II^a^	21.1% (4/19)	1.00 (reference)	--	1.00 (reference)	--	1.00 (reference)	--
III-IV	76.2% (16/21)	12.0 (2.70-53.3)	0.001*	9.63 (1.05-88.76)	0.046*	41.01 (3.62-464.91)	0.003*
Lymph node metastasis							
No	15.4% (2/13)	1.00 (reference)	--	1.00 (reference)	--		
Yes	66.7% (18/27)	11.0 (2.00-60.5)	0.006*	20.92 (1.20-365.55)	0.037*		
Number of lymph node metastasis							
0	15.4% (2/13)	1.00 (reference)	--				
1-4 s	60.0% (9/15)	8.25 (1.33-51.2)	0.024*				
≥5	75.0% (9/12)	16.5 (2.25-121.2)	0.006*				

† In multivariate Model 1, CD44 and CD24 expression were considered as two variables.

‡ In multivariate Model 2, CD44 and CD24 expression were combined as one variable to show different combinations.

^a^ Two control patients with TNM Stage 0 were grouped into Stage I-II.

Note: OR, odds ratio; aOR, adjusted odds ratio.

*P<0.05.

In multivariate model 1, when CD44 and CD24 expression and age were forced into the model, only gender (male vs. female, aOR = 39.64, 95% CI: 1.85-848.44, *P* = 0.019), TNM stage (Stage III-IV vs. I-II, aOR = 9.63, 95% CI: 1.05-88.76, *P* = 0.046), and lymph node metastasis (Yes vs. No, aOR = 20.92, 95% CI: 1.20-365.55, *P* = 0.037) achieved a significance level allowing them to be retained in the multivariate logistic regression model. However, after adjusting for other risk factors, the association of CD44 expression (aOR = 0.66, 95% CI: 0.10-4.26, *P* = 0.658) and CD24 expression (aOR = 0.09, 95% CI: 0.01-1.35, *P* = 0.081) with gastric cancer recurrence were still not significant.

In multivariate model 2, when combined CD44/CD24 expression and age were forced into the model, only gender (male vs. female, aOR = 21.59, 95% CI: 1.24-377.26, *P* = 0.035) and TNM stage (Stage III-IV vs. I-II, aOR = 41.01, 95% CI: 3.62-464.91, *P* = 0.003) achieved a significance level allowing them to be retained in the multivariate logistic regression model. Compared to patients with combined expression of CD44-/CD24-, the adjusted ORs for CD44-/CD24+, CD44+/CD24-, and CD44+/CD24+ were 0.60 (95% CI: 0.03-10.67), 1.61 (95% CI: 0.15-17.36), and 0.06 (95% CI: 0.002-2.31), respectively. After adjusting for other risk factors, the association of CD44/CD24 expression with gastric cancer recurrence was still not significant.

## Discussion

Evaluation of the correlation of CD44/CD24 expression with recurrent gastric cancer revealed no differences in risk of gastric cancer recurrence between CD44+ patients and CD44- patients. Although CD24+ patients had a higher likelihood of gastric cancer recurrence than CD24- patients, significance was not demonstrated. Our study results suggest that these molecules do not appear to be clinically useful for prediction of gastric carcinoma recurrence after curative resection.

The role of CD44 and CD24 expression in gastric carcinoma has been explored for nearly thirty years. Numerous studies have focused on the diagnostic and prognostic significance of CD44 expression in human tumors, especially gastric cancer. In 1982, CD44 was identified as a surface glycoprotein and a lymphocyte homing receptor found on lymphoid and epithelial cells [8]; its main function on lymphocytes is mediating interaction with the endothelium, but its function on epithelial cells is not entirely understood [9]. The CD44 proteins belong to a family of type I transmembrane glycoproteins that are encoded by a single, highly conserved gene located on the short arm of chromosome 11 in humans; two molecular sizes have been identified: low *M*_r_ CD44 (80–90 x 10^3^) is expressed in lymphoid tissue and high *M*r CD44 (130–160 x 10^3^) is expressed in tumor cells and keratinocytes [10]. Some aggressive tumors are reported to be associated with the expression of CD44. Overexpression of CD44, defined by apparently increased expression of CD44 protein, has been linked to poor prognosis with tumor progression and metastatic potential in several human malignancies, including gastric cancer [7], colorectal cancer, breast cancer [5,6], uterine cancer, ovarian cancer, bladder cancer, lung cancer, hematopoietic malignancies, and gliomas [11]. A retrospective study of 100 patients with gastric cancer evaluated the expression of CD44 and its prognostic importance, concluding that this cell adhesion molecule is highly expressed in gastric adenocarcinoma [7]. In that study, expression of CD44 correlated with a poor prognosis in patients with the intestinal type of gastric adenocarcinoma. These investigators suggested that CD44 could be utilized as a prognostic marker for this group of patients. In this study, the proportion of lymph node metastasis was significantly higher in the GC study group than that in the control group and the proportion of patients with 5 or more metastatic lymph nodes was also significantly higher in the study group than in the control group. However, while CD44 was definitely linked with GC in our study and prognostic to some degree, we could not verify its predictive capability in terms of GC recurrence after tumor resection.

CD24, a mucin-type GPI-linked cell surface molecule on human neutrophils and pre-B lymphocytes, plays an important role in the margination and adhesion of cells under shear force of blood flow [11]. Positive CD24 expression is found to occur in a subset of GC and to correlate with lymphatic invasion, blood vessel invasion and poor survival. The clinicopathological significance of CD24 expression in human gastric adenocarcinoma was evaluated by Chou and colleagues, who concluded that cytoplasmic expression of CD24 was associated with invasiveness and poorer prognosis and can serve as a novel target for prognostic prediction and adjuvant treatment of patients with diffuse-type gastric adenocarcinoma after tumor resection [12]. Further studies are needed to investigate other combinations of adhesion molecules.

In the present study, CD44 and CD24 expression, independently and in combination, were not associated with GC recurrence. Carcinoma of the intestinal type are more frequently CD44s and CD44v6 positive than carcinomas of the diffuse type, and the importance of subclassifying tumor types in investigations of CD44 in human cancer has been demonstrated [13]. When cytoplasmic CD24 expression was studied in diffuse-type gastric adenocarcinoma, it was shown to be associated with invasiveness, lymph node metastasis and poorer prognosis, but not specifically associated with recurrence after tumor resection; however, no significant differences were seen in tumor stage or lymph node metastasis between mixed-type GC with or without CD24 expression [12]. Although there is no ideal cross-comparison between different histological grading systems, we did classify GC according to the WHO histological classification and Lauren’s classification in the present study. We had no cases of lymphoepithelial-like carcinoma or mucosa-associated lymphoid tissue (MALT) lymphoma.

Another possible explanation of lack of significant associations between CD44 and CD24 and recurrence may be the particular isoform of CD44 identified in our study. Up to seven molecular forms have varying functional roles in vivo such as having different abilities to bind hyaluronate, while their most important common feature is their expression on tumor cells and correlation with metastases [10]. These forms can be identified more readily by sequencing mRNAs and only a few are identified by protein analysis. The distribution of CD44 and CD24 may also differ and cellular sites in normal tissue have only been identified in animal models, and are not confirmed in normal human tissue. However, larger forms are found as minor components in normal tissue and low *M*_r_ forms are associated with lymphoid cell types in tissue [14]. The function of CD44/CD24 is complex and changes in CD44, in particular, have been noted in carcinogenesis, including gene expression modulation, splicing of RNA and altered glycosylation [13]. Any of the above factors may account for the apparent lack of association with recurrence in our study. Clearly, more research is needed.

Molecular pathways involved in GC have been identified over the past twenty years [15]. Current investigations of molecular markers for gastric progenitor cells and gastric stem cells may hold promise for learning more about GC, its progression and propensity for recurrence, and eventually for treatment applications. This possibility is primarily because gastric tissue, as well as intestinal tissue undergoes constant epithelial cell replacement and because stem cells and progenitor cells play important roles in the renewal of gastric glands and in epithelial repair following tissue injury [16]. Zhang et al. [17] examined CD44 and CD24 in gastric cells lines, AGS and gastric cancer tissues and identified the tumorigenic properties, self-renewal and differentiated progeny in CD44 + CD24+ and CD44-CD24- cell populations. As few as 200 of the positive combination of cells injected into mice generated tumors in 50%, while many thousands more negative combined cells were needed to form a tumor in a second group of mice, suggesting that the subpopulation of CD44 + CD24+ gastric cell lines (AGS tumor cell lines) is GC stem cells [17]. In protein studies, CD24, which is associated with tumor metastasis, and Galectin-1 expression, which is associated with immune response and tumor progression, were studied in GC patients, giving particular attention to staining intensity and clinicopathologic variables; the researchers concluded that these proteins were independent prognostic indicators of poor survival (though not specifically associated with recurrence) and could be useful as therapeutic targets [18]. Since we are not yet studying the unique progenitor and stem cell populations in human models, we should continue exploring tumor-specific protein expression that may be associated with GC and recurrent GC, seeking to find a molecular basis for this globally prevalent disease and new paths to diagnosis, prognosis and treatment.

### Limitations

This study has several limitations, with the main limitation being its retrospective nature and limited sample size. In addition, the functional significance of CD44+/CD24- cells is still unclear. Their importance in metastasis, and thus in disease recurrence and gastric cancer mortality, is not yet well understood. This small retrospective series served as an initial study to investigate the potential association between CD44 and CD24 and gastric cancer recurrence, but a study with a larger sample is needed to confirm our preliminary results. We must also acknowledge that we were unable to confirm intratumor heterogeneity because there were only limited numbers of surgically resected specimens of the recurrent or metastatic tumors for repeat CD44/CD24 staining, which may be considered in future studies.

## Conclusion

In conclusion, neither individual expression of CD24 and CD44 nor combined expression of CD44/CD24 was associated with recurrence of gastric carcinoma. Since adhesion molecules CD44 and CD24 do not appear to be clinically useful for prediction of gastric carcinoma recurrence after curative resection, future research is warranted to both confirm these results and to investigate other possible biomarkers.

## Competing interests

The authors declare that they have no competing interests.

## Authors’ contributions

CSY, CMOY, and CWL performed majority of experiments; CMOY and YHC collected all human material; CCL reviewed pathologies; CSL analyzed data; CSY and CMOY designed the study and wrote the manuscript. All authors read and approved the final manuscript.

## Pre-publication history

The pre-publication history for this paper can be accessed here:

http://www.biomedcentral.com/1471-230X/12/95/prepub
